# High dimensional phase resetting curves and their use in predicting network dynamics

**DOI:** 10.1186/1471-2202-12-S1-P307

**Published:** 2011-07-18

**Authors:** Sorinel A Oprisan, Robert Raidt, Andrew Smith

**Affiliations:** 1 Department of Physics and Astronomy, College of Charleston, SC 29624, USA

## 

**Figure 1:**
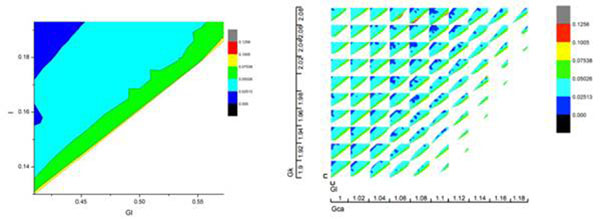
A five-dimensional plot of the first DST coefficient of type 2 PRC for ML model neuron. The two-dimensional color-coded plot versus leak and external bias at constant calcium (Gca = 1 mS/cm^2^) and potassium (Gk = 1.9 mS/cm^2^).

Neural networks continuously correct their response to environmental stimuli in order to allow animals to better fit their niches. External stimuli, such as light or temperature, reset or entrain the endogenous rhythms such as those generated by the circadian clock. Many neurons are endogenous oscillators, i.e., they generate a regular train of action potentials, in the absence of any inputs. Brief perturbations of neural oscillators, such as external current pulses, chemical or electrical synaptic inputs, or exposure to neuromodulators, can reset neural activity and might explain how the same neural network can discriminate among multiple stimuli, how neurons correlate input signals, how the firing frequency of an individual neuron changes, or how cellular mechanisms are controlled by external stimuli. From a theoretical perspective, modeling stimulus-induced resetting was done using phase-coupled oscillators. We use in this study an alternative approach to resetting and entrainment in neural networks based on phase resetting curve (PRC). The main advantage of this method is that it can be applied to heterogeneous networks and is not limited to small amplitude and short duration (pulsatile) couplings among neurons. Moreover, the PRC method can be used even without explicitly knowing the model equations by simply tabulating the transient changes in the firing rate of an isolated neuron subject to an external stimulus. The ultimate purpose of studying PRCs is to use them in predicting the firing patterns of neuronal networks. The advantage of the PRC method over a detailed (and always incomplete) biophysically model of a neurons is that a) it is readily available from simple electrophysiological data and, 2) computation with such a neural network is much faster. In this study, we were concerned with the quantitative effect different ionic current shave on the shape of the PRC. A Morris-Lecar model neuron was used with the purpose of numerically extracting the PRC. Model parameters were tuned such that we investigated both type 1 and type 2 excitability and PRCs were extracted for different bias current, calcium, potassium, and leak conductance. In order to make a quantitative comparison regarding the influence of different parameters on the shape of the PRC, we actually stored a limited number of coefficients of the discrete sine transform (DST) of each PRC (see Fig. 1). The multidimensional plot made of 10x10 color-coded two-dimensional plots of the first DST coefficient for variable leak (Gl) and external bias (I) and with constant calcium (Gca) and potassium (Gk) conductances (left panel of Fig.1). We investigated the relationship among different DST coefficients in order to minimize the required amount of storage per each PRC while retaining acceptable accuracy. The PRCs were subsequently used to predict the firing patterns for different combinations of control parameters.

